# Atherosclerosis-Related Circulating miRNAs as Novel and Sensitive Predictors for Acute Myocardial Infarction

**DOI:** 10.1371/journal.pone.0105734

**Published:** 2014-09-03

**Authors:** Feng Wang, Guangwen Long, Chunxia Zhao, Huaping Li, Sandip Chaugai, Yan Wang, Chen Chen, Dao Wen Wang

**Affiliations:** The Institute of Hypertension and Department of Internal Medicine, Tongji Hospital, Tongji Medical College, Huazhong University of Science and Technology, Wuhan, People's Republic of China; University Heart Center Freiburg, Germany

## Abstract

**Background:**

The dysregulated expressions of circulating miRNAs have been detected in various cardiovascular diseases. In our previous experiments, the altered expressions of circulating miRNA-21-5p, miRNA-361-5p and miRNA-519e-5p were confirmed in patients with coronary atherosclerosis by miRNA microarrays. However, the expression levels of these circulating miRNAs in the early phase of acute myocardial infarction (AMI) are still unknown. In the present study, our aims were to examine the expressions of circulating miR-21-5p, miR-361-5p and miR-519e-5p in AMI patients, and assess their clinical applications for diagnosing and monitoring AMI.

**Results:**

Two different cohorts were enrolled in this study. The first cohort included 17 AMI patients and 28 healthy volunteers, and the second cohort included 9 AMI patients, 9 ischemic stroke patients, 8 patients with pulmonary embolism, and 12 healthy volunteers. Quantitative real-time PCR and ELISA assays were preformed to detect the concentrations of plasma miRNAs and cardiac troponin I (cTnI), respectively. The results showed that the plasma levels of miR-21-5p and miR-361-5p were significantly increased in AMI patients, whereas the concentration of circulating miR-519e-5p was reduced. Interestingly, the levels of these circulating miRNAs correlated with the concentrations of plasma cTnI. Receiver operating characteristic (ROC) analysis revealed that these three circulating miRNAs had considerable diagnostic accuracy for AMI with high values of area under ROC curve (AUC). Importantly, combining the three miRNAs significantly increased the diagnostic accuracy. Furthermore, cell experiments demonstrated that these plasma miRNAs may originate from injured cardiomyocytes induced by hypoxia. In addition, the levels of all the three circulating miRNAs in ischemic stroke (IS) and pulmonary embolism (PE) were elevated, whereas the decreased level of plasma miR-519e-5p was only detected in AMI. ROC analysis demonstrated that circulating miR-519e-5p may be a useful biomarker for distinguishing AMI from other ischemic diseases.

**Conclusions:**

Circulating miRNAs may be novel and powerful biomarkers for AMI and they could be potential diagnostic tool for AMI.

## Background

Due to the high morbidity and mortality, acute myocardial infarction is the worst disease of acute myocardial ischemia. So, an early and correct diagnosis is essential for controlling the development of AMI and initiating the appropriate therapy to potentially reduce the mortality rate and improve prognosis [1]. Blood troponins, cardiac myoglobin and creatine kinase-MB are used as biomarkers for diagnosing AMI [2], and plasma cTnI is widely used in clinical practice as the gold standard for diagnosing AMI [3]. However, elevated plasma cTnI was not only observed in certain cardiac ischemic injury, but also in some other diseases such as severe heart failure, atrial fibrillation, chronic kidney disease, severe sepsis, septic shock etc [4], [5], [6]. Therefore, it is necessary to find novel and effective biomarkers for early and accurate diagnosis of AMI.

MicroRNAs (miRNAs) are endogenous, small noncoding RNAs that play crucial roles in regulation of gene expression through binding to the 3′ UTR of target mRNA at the post-transcription processing steps [7]. In recent years, miRNAs have been proven to play important roles in a variety of physiological and pathological processes, such as development, metabolism, cellular differentiation, proliferation, cell death and stress response [7], [8], [9]. Some studies demonstrated that miRNAs are abundantly present in plasma/serum in a remarkably stable form and can be detected by real-time PCR assays [10], [11], [12], and the expression profiling of circulating miRNAs may change in various diseases. These results suggest that circulating miRNAs could serve as potentially useful candidates for diagnostic and other clinical applications. Previous studies demonstrated that circulating miRNAs could be used as novel and potential biomarkers for the diagnosis and prognosis of diseases, such as various cancers, heart disease, pregnancy, diabetes, psychosis, and various infectious diseases [13], [14], [15], [16], [17], [18], [19]. Recently, circulating miRNAs have been detected in a variety of cardiovascular diseases, and they can be used as biomarkers for improving the diagnostic accuracy of cardiovascular diseases [20], [21], [22], [23], [24], as well as serving as predictors for cardiovascular events [25].

In our previous experiments, the altered expressions of circulating miR-21-5p, miR-361-5p and miR-519e-5p were observed in patients with coronary artery disease (CAD) by miRNA microarray screening (manuscript under revision). The levels of miR-21-5p and miR-361-5p were increased approximately 4.17-fold (p = 0.0008) and 127-fold (p = 0.004) in coronary atherosclerosis patients compared to healthy controls, respectively, whereas miR-519e-5p was decreased 35% (p<0.01) in plasma of coronary atherosclerosis patients. However, the expression pattern of circulating miR-21-5p, miR-361-5p and miR-519e-5p in AMI remains unknown. In this study, we assessed the hypothesis that the levels of these three miRNAs may change during myocardial infarction. Our aims were to investigate the dynamic expression of circulating miR-21-5p, miR-361-5p, and miR-519e-5p in early phase of AMI, and confirm the source of these circulating miRNAs. In addition, we also assessed the abilities of the three circulating miRNAs for identifying and evaluating AMI.

## Results

### The pattern of circulating miRNAs and cTnI levels in patients with AMI

**Figure 1:**
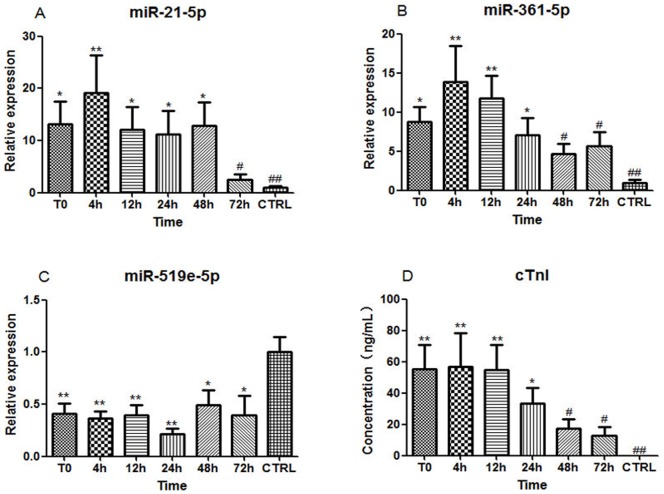
The expression levels of circulating miRNAs and cTnI in AMI patients. The dynamic expressions of circulating miRNAs and plasma cTnI in AMI patients at a time course, The initial time (T0) was 12.4±1.5 hours after the onset of AMI symptoms. (A) miR-21-5p; (B) miR-361-5p; (C) miR-519e-5p; (D) cTnI. Data are presented as mean ± SEM, *p<0.05, **p<0.01 versus healthy control; ^#^p<0.05, ^##^p<0.01 versus peak level.

**Figure 2:**
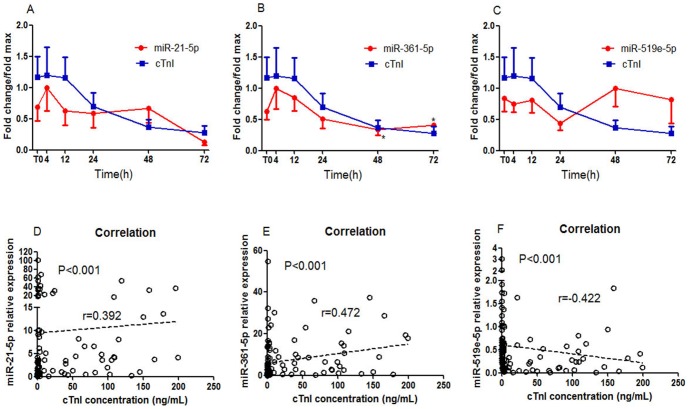
The expression pattern and the correlations between circulating miRNAs and plasma cTnI in AMI patients. (A) the expression trend of circulating miR-21-5p and plasma cTnI in AMI patients; (B) the expression trend of circulating miR-361-5p and plasma cTnI; (C) the expression trend of circulating miR-519e-5p and plasma cTnI; (D) The relationship between circulating miR-21-5p and plasma cTnI in AMI patients; (E) The relationship between miR-361-5p and cTnI; (F) The relationship between miR-519e-5p and cTnI. Data are presented as mean ± SEM, *p<0.05, **p<0.01 versus peak level.

To investigate the expression patterns of circulating miRNAs in early AMI, 17 patients with AMI and 28 healthy volunteers without evidence for CAD were enrolled in the first cohort. Six blood samples were collected from each AMI patient at various time points as described in the Methods. The clinical characteristics of this cohort are summarized in Table 1 and Table S1 in File S1. The initial time (T0) was 12.4±1.5 hours after the onset of AMI symptoms. The results showed that the expressions of circulating miR-21-5p and miR-361-5p were significantly elevated, whereas the plasma level of miR-519e-5p was remarkably reduced in AMI patients compared to healthy volunteers (Fig. 1). Elevated plasma miR-21-5p and miR-361-5p achieved their peak at 4h after T0 (19.1-fold and 13.8-fold, respectively), whereas plasma miR-519e-5p exhibited its lowest expression at 24h after T0 (approximately 78.4% decrease). Plasma cTnI concentrations were simultaneously measured in the same plasma samples by ELISA assays. The results showed that plasma levels of cTnI were remarkably increased in AMI patients compared to healthy volunteers, and it achieved a 2448.2-fold peak at 4 h after T0 (Fig. 1D). Furthermore, the expression patterns of these three circulating miRNAs were compared with plasma cTnI. Interestingly, circulating miR-21-5p and miR-361-5p (especially miR-361-5p) exhibited similar trends to plasma cTnI in the early phase of AMI (Fig. 2A and 2B). Both of them achieved their peak expression at 4 h after T0, and then gradually declined in the following hours, whereas circulating miR-519e-5p remained low until 48 h in AMI group (Fig. 2C).

**Table 1:** The baseline clinical characteristics of the first cohort.

Characteristics	Total participants (n = 45)	AMI patients (n = 17)	Healthy volunteers (n = 28)	P value
Age (years)	54±11	52±11	58±11	0.345
Male/female (n/n)	24/21	12/5	12/16	0.071
Smoking, n (%)	14 (31.1)	5 (17.8)*	9 (52.9)	0.014
Hypertension, n (%)	13 (28.9)	4 (14.4)**	9 (52.9)	0.006
Diabetes, n (%)	1 (2.2)	0 (0.0)	1 (5.9)	0.194
SBP (mmHg)	127.4±19.9	134.2±25.9	122.9±13.7	0.078
DBP (mmHg)	76.7±11.8	73.7±10.1	81.1±12.9	0.147
WBC (×10^9^/L)	7.6±2.73	9.7±3.14**	6.4±1.34	<0.001
BUN (mmol/L)	5.89±2.31	6.16±3.0	5.73±1.83	0.329
Fasting glucose (mmol/L)	6.49±1.52	6.86±1.68	6.24±1.38	0.662
TC (mmol/L)	4.28±1.07	4.42±1.32	4.19±0.89	0.100
TG (mmol/L)	1.37±0.95	1.47±0.61	1.30±1.11	0.257
HDL (mmol/L)	1.11±0.23	1.03±0.21	1.16±0.24	0.383
LDL (mmol/L)	2.36±0.79	2.43±0.68	2.33±0.86	0.452

SBP, systolic blood pressure; DBP, diastolic blood pressure; WBC, white blood cell; BUN, blood urea nitrogen; TC, total cholesterol; TG, Triglyceride; HDL, high-density lipoprotein; LDL, low-density lipoprotein; comparison between AMI patients and healthy persons,

*p<0.05,

**p<0.01 versus healthy control. (Data are presented as mean ± SD).

In order to confirm whether the levels of these miRNAs in plasma are associated with plasma cTnI in AMI patients, correlation analysis was performed. The results showed that miR-21-5p (r = 0.392) and miR-361-5p (r = 0.472) exhibited significant positive correlation with cTnI, whereas a negative relationship (r = −0.422) was observed between plasma miR-519e-5p and cTnI (Fig. 2D–2F).

**Figure 3:**
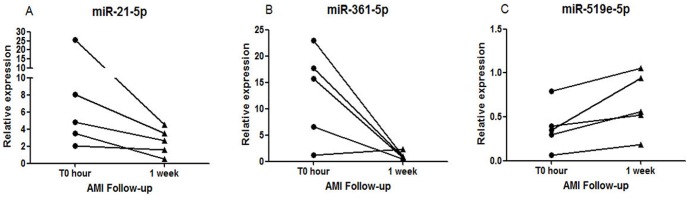
The follow-up expression of circulating miRNAs. Blood samples from 5 AMI patients who received PCI treatment were collected at 1 week after admitted to hospital. The follow up expressions of circulating (A) miR-21-5p, (B) miR-31-5p, and (C) miR-519e-5p in AMI patients after receiving medical treatment. Data are presented as mean ± SEM.

A follow-up investigation was performed to determine whether the plasma miRNAs levels in AMI patients are associated with prognosis after receiving appropriate treatments. Among the 17 AMI patients in the first cohort, 5 patients received emergency percutaneous coronary intervention (PCI), and plasma samples were collected at 1 week after admission to the hospital. As shown in Figure 3, the plasma levels of miR-21-5p (Fig. 3A) and miR-361-5p (Fig. 3B) declined and circulating level of miR-519e-5p (Fig. 3C) gradually increased towards their baseline values after 1 week. These observations indicated that the plasma levels of miR-21-5p, miR-361-5p and miR-519e-5p may gradually return to normal levels (over 1 week) after receiving appropriate treatments.

### The cellular origin of circulating miRNAs in AMI

**Figure 4:**
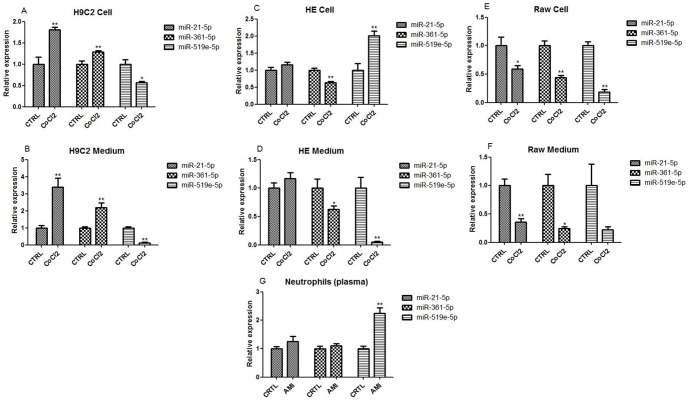
The expressions of miRNAs in cardiomyocytes, endothelium, macrophages, and their medium in condition of hypoxia. Cobalt chloride was used for inducing hypoxia. After 24 hours treatment, cells and medium were collected for detecting the expression of miRNAs, respectively. (A) The expression of miRNAs in cardiomyocytes (H9c2) under hypoxia; (B) The expression of miRNAs in medium of cardiomyocytes (H9c2) under hypoxia; (C) The expression of miRNAs in endothelium (HE) under hypoxia; (D) The expression of miRNAs in medium of endothelium (HE) under hypoxia; (E) The expression of miRNAs in macrophages (Raw 264.7) under hypoxia; (F) The expression of miRNAs in medium of macrophages (Raw 264.7) under hypoxia; (G) The expressions of circulating miRNAs in peripheral blood neutrophils isolated from AMI patients and healthy subjects. Data are presented as mean ± SEM, *p<0.05, **p<0.01 versus control.

To investigate the source of these circulating miRNAs in AMI, cobalt chloride (CoCl_2_) was used to induce hypoxia in cardiomyocytes (H9c2), endothelial cells (HUVEC), and macrophages (Raw 264.7). After 24 hours of treatment with CoCl_2_, cells and medium were collected to examine the expression of miRNAs, respectively. The results showed that the expression of miR-21-5p and miR-361-5p were increased in both cardiomyocytes (Fig. 4A) and their medium (Fig. 4B) following treatment with CoCl_2_, whereas the miR-519e-5p level was significantly decreased. Interestingly, the dynamic expressions of the three miRNAs in cardiomyocytes and their medium were consistent with the expressions of circulating miRNAs in serum of AMI patients. Furthermore, hypoxia could remarkably reduce the level of miR-361-5p both in endothelial cells (Fig. 4C) and their medium (Fig. 4D), whereas the change in miR-21-5p expression was not observed. Interestingly, the expression pattern of miR-519e-5p in endothelium and supernatant were opposite under hypoxic conditions. Although the level of miR-519e-5p was increased in endothelial cells, the concentration of extracellular miR-519e-5p was significantly reduced in the medium. In addition, the expression of all the three miRNAs in macrophages (Fig. 4E) and their medium (Fig. 4F) were markedly reduced under hypoxia. These observations indicated that cardiomyocytes may be the main source of these circulating miRNAs during the condition of anoxic injury.

In addition, peripheral blood neutrophils were isolated from all the 9 AMI patients and 5 healthy subjects to examine the expression of the three miRNAs in circulating neutrophils. The expressions of miR-21-5p and miR-361-5p did not change in peripheral blood neutrophils between AMI patients and healthy volunteers, but the expressions of miR-519e-5p in neutrophils from AMI patients was remarkably increased (Fig. 4G). These results raised the intriguing possibility that the reduction of circulating miR-519e-5p in AMI may result from uptake by peripheral blood neutrophils.

### The expression pattern of circulating miRNAs in other ischemic diseases

**Figure 5:**
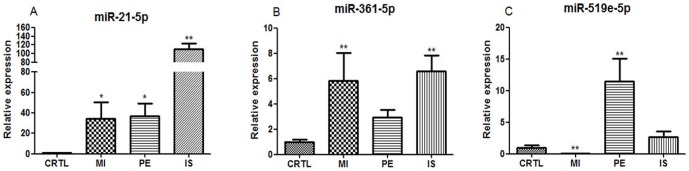
The expressions of circulating miRNAs in patients with AMI, PE, and IS, respectively. AMI, acute myocardial infarction; PE, pulmonary embolism; IS, ischemic stroke; (A) The expression of circulating miR-21-5p in patients with AMI, PE, and IS, respectively; (B) The expression of circulating miR-361-5p in patients with AMI, PE, and IS, respectively; (C) The expression of circulating miR-519e-5p in patients with AMI, PE, and IS, respectively; Data are presented as mean ± SEM, *p<0.05, **p<0.01 versus control.

In order to evaluate whether the three miRNAs are specific to cardiac ischemia, 9 ischemic stroke patients, 8 patients with pulmonary embolism, 9 AMI patients, and 12 healthy volunteers were recruited in the second cohort. The baseline clinical characteristics of this cohort are presented in Table 2. MiRNAs quantitative analysis showed that the levels of circulating miR-21-5p (Fig. 5A) and miR-361-5p (Fig. 5B) were significantly increased in all three ischemic diseases compared to healthy volunteers. Interestingly, the expression of plasma miR-519e-5p was increased in IS and PE, whereas its level was remarkably reduced in plasma of AMI patients (Fig. 5C). These data suggested that circulating miR-519e-5p may be an appropriate biomarker for discriminating AMI from cerebral infarction and pulmonary embolism.

**Table 2:** The baseline clinical characteristics of the second cohort.

Characteristics	Control (n = 12)	AMI (n = 9)	PE (n = 8)	IS (n = 9)	P value
Age (years)	57±8	59±9	42±9**	58±7	<0.001
Male/female (n/n)	5/7	6/3	4/4	7/2	0.355
Smoking, n (%)	2(16.7)	2(22.2)	1(12.5)	7(77.8)**	0.008
Hypertension, n (%)	6 (50)	3 (33.3)	2 (25)	5 (55.6)	0.528
Diabetes, n (%)	3 (25)	0 (0)	1 (12.5)	1 (11.1)	0.412
SBP (mmHg)	129.3±20.8	120.7±14.8	122.6±17.5	150.7±20.2*	0.007
DBP (mmHg)	78.7±9.2	73.8±9.9	80.0±11.9	89.6±10.0*	0.018
WBC (×10^9^/L)	6.7±1.29	8.3±3.44	7.5±3.60	5.6±1.70	0.170
BUN (mmol/L)	5.41±1.50	5.87±2.27	5.26±2.99	6.23±2.12	0.778
Fasting glucose (mmol/L)	6.09±2.19	5.76±1.33	6.06±0.95	6.07±2.03	0.980
TC (mmol/L)	4.20±1.10	3.88±0.88	3.83±0.89	3.44±1.12	0.444
TG (mmol/L)	1.49±0.83	2.34±1.53*	1.04±0.50	1.30±0.53	0.030
HDL (mmol/L)	1.04±0.30	0.89±0.19	0.93±0.38	0.81±0.26	0.410
LDL (mmol/L)	2.44±0.32	2.12±0.70	2.42±0.67	2.13±0.87	0.810

AMI, acute myocardial infarction; PE, pulmonary embolism; IS, ischemic stroke; SBP, systolic blood pressure; DBP, diastolic blood pressure; WBC, white blood cell; BUN, blood urea nitrogen; TC, total cholesterol; TG, Triglyceride; HDL, high-density lipoprotein; LDL, low-density lipoprotein; comparison between AMI patients and healthy persons,

*p<0.05,

**p<0.01 versus healthy control. (Data are presented as mean ± SD).

### Evaluation of circulating miRNAs as sensitive and potential predictors for AMI

To investigate the possibility that these miRNAs may serve as new and potential biomarkers for AMI, ROC analysis was performed in above two recruited cohorts.

**Figure 6:**
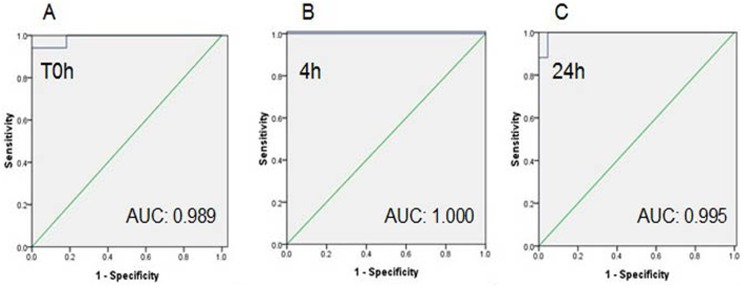
Diagnostic value of the combination plasma miRNAs in AMI patients from first cohort. The ROC curves of combining miR-361-5p, miR-21-5p and miR-519e-5p at T0h (A), 4 h (B) and 24 h (C), respectively.

In the first cohort, because T0 was the first time point of sample collection, and 4 h and 24 h were the time points of maximum/minimum miRNA expression levels, the data of circulating miRNAs levels at T0, 4 h, and 24 h were selected for further evaluation. As shown in Table 3, ROC curve analysis of miR-21-5p and miR-361-5p exhibited strong differentiation power between AMI patients and healthy volunteers at various time points in the early phase of AMI, whereas miR-519e-5p showed a moderate differentiation power (Fig. S1 in File S1). However, the AUC of plasma cardiac troponin I (1.000; 95% confidence interval 1.000–1.000) was higher than any of the three miRNAs at T0, 4 h, and 24 h. Interestingly, the combination of three miRNAs resulted in a much higher AUC value of 0.989 (95% confidence interval 0.000–1.000), 1.000 (95% confidence interval 0.000–1.000) and 0.995 (95% confidence interval 0.000–1.000) at T0, 4 h and 24 h, respectively (Fig. 6).

**Table 3:** Diagnostic value of circulating miRNAs and cTnI in AMI patients.

Time point	miRNAs	AUC	95% Cl	P value
**T0**	miR-21-5p	0.949	0.872–1.000	P<0.001
	miR-361-5p	0.881	0.777–0.985	P<0.001
	miR-519-5p	0.798	0.663–0.934	P = 0.001
	**combined score**	0.989	0.000–1.000	P<0.001
	cTnI	1.000	1.000–1.000	P<0.001
**4 h**	miR-21-5p	0.947	0.000–1.000	P<0.001
	miR-361-5p	0.883	0.777–0.989	P<0.001
	miR-519-5p	0.801	0.668–0.934	P = 0.002
	**combined score**	1.000	0.000–1.000	P<0.001
	cTnI	1.000	1.000–1.000	P<0.001
**24 h**	miR-21-5p	0.791	0.655–0.927	P = 0.001
	miR-361-5p	0.838	0.716–0.961	P<0.001
	miR-519-5p	0.908	0.818–0.997	P<0.001
	**combined score**	0.995	0.000–1.000	P<0.001
	cTnI	1.000	1.000–1.000	P<0.001

Combined score, the combination of miR-21-5p, miR-361-5p and miR-519e-5p; cTnI, cardiac troponin; AUC, area under the ROC curve; 95% Cl, 95% confidence interval.

The diagnostic value of the three circulating miRNAs for AMI were further verified in the second cohort, meanwhile the ability of circulating miR-519e-5p for distinguishing AMI from PE and IS was also evaluated. The data are summarized in Table 4. Circulating miR-21-5p, miR-361-5p and miR-519e-5p exhibited a powerful differentiation value for AMI patients and healthy volunteers, and the combination of the three miRNAs yielded the highest AUC value of 1.000 (95% CI 1.000–1.000).

**Table 4:** Diagnostic value of circulating miRNAs among different ischemic diseases.

	miRNAs	AUC	95% Cl	P value
AMI vs. Control	miR-21-5p	0.981	0.000–1.000	P<0.001
	miR-361-5p	0.907	0.000–1.000	P = 0.002
	miR-519-5p	0.944	0.000–1.000	P = 0.001
	**Combined score**	1.000	1.000–1.000	P<0.001
PE vs. Control	miR-21-5p	1.000	1.000–1.000	P<0.001
	miR-361-5p	0.875	0.000–1.000	P = 0.005
	miR-519-5p	0.927	0.000–1.000	P = 0.002
	**Combined score**	1.000	1.000–1.000	P<0.001
IS vs. Control	miR-21-5p	1.000	1.000–1.000	P<0.001
	miR-361-5p	0.969	0.000–1.000	P = 0.001
	miR-519-5p	0.708	0.472–0.945	P = 0.123
	**Combined score**	1.000	1.000–1.000	P<0.001
AMI vs. PE	miR-21-5p	0.583	0.298–0.869	P = 0.564
	miR-361-5p	0.444	0.157–0.732	P = 0.700
	miR-519-5p	1.000	1.000–1.000	P = 0.001
AMI vs. IS	miR-21-5p	0.889	0.000–1.000	P = 0.005
	miR-361-5p	0.617	0.332–0.902	P = 0.402
	miR-519-5p	1.000	1.000–1.000	P<0.001
IS vs. PE	miR-21-5p	0.917	1.000–1.000	P = 0.004
	miR-361-5p	0.806	0.549–1.000	P = 0.034
	miR-519-5p	0.819	0.000–1.000	P<0.027

AMI, acute myocardial infarction; PE, pulmonary embolism; IS, ischemic stroke; Combined score, the combination of miR-21-5p, miR-361-5p and miR-519e-5p; AUC, area under the ROC curve; 95% Cl, 95% confidence interval.

However, the elevated miR-21-5p and miR-361-5p were not specific for diagnosing AMI, their levels were also significantly increased in patients with PE and IS. ROC analysis revealed that elevated miR-21-5p and miR-361-5p also showed significantly higher diagnostic value for PE and IS. It is noteworthy that circulating miR-519e-5p exhibited a lower level in AMI patients, which was opposite to its expression in other ischemic diseases. Circulating miR-519e-5p may be a potent biomarker for distinguishing AMI from PE and IS, with the AUC of 1.000 (95% CI 1.000–1.000).

Taken together, all these data suggested that these three circulating miRNAs (miR-21-5p, miR-361-5p, and miR-519e-5p), especially miR-519e-5p, may be used as sensitive and independent predictors for AMI.

### The impact of heparin on circulating miRNAs expression

**Figure 7:**
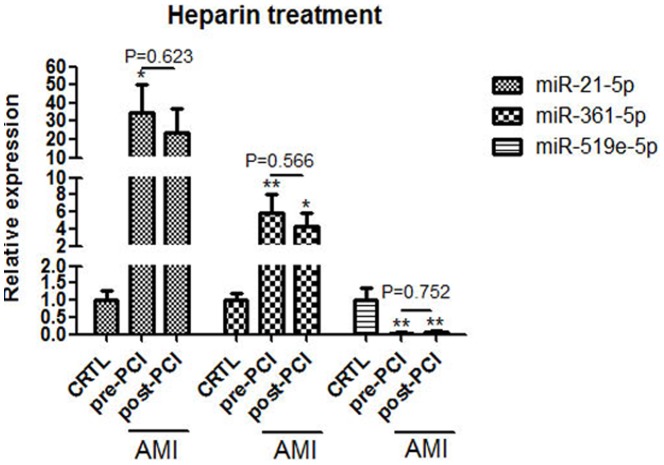
The impact of heparin on circulating miRNAs' expressions. CRTL, control; Data are presented as mean ± SEM, *p<0.05, **p<0.01 versus control.

A previous study reported that heparin selectively affects the expression of circulating miRNAs. To investigate whether the expressions of these three miRNAs would be affected by heparin after the cardiac catheterization procedure, two blood samples were collected from all the 9 AMI patients in the second cohort before and after PCI. The average amount of heparin was 5889±675 IU. As shown in Fig. 7, there were no statistically significant differences in plasma levels of the three circulating miRNAs between pre-PCI and post-PCI.

## Discussions

Based on their tissue specificity, rapid release kinetics and stability in plasma, circulating miRNAs have been proposed as potentially useful and novel biomarkers for detecting various cardiovascular diseases [26], including heart failure [21], myocardial infarction [22], coronary artery disease [23], hypertension [17], pulmonary arterial hypertension [27] etc. Circulating miRNAs can not only be used as novel biomarkers for diagnosing cardiovascular diseases, but also assist in extracellular communication for promoting the communication between different cells/tissues through microparticles or HDL [28], [29], [30], [31].

Acute myocardial infarction, with high morbidity and mortality, has always attracted more attention in the field of cardiovascular medicine. Previous studies demonstrated that numerous circulating miRNAs are involved in AMI, including cardiac-specific miRNAs (miR-208 [32], miR-499 [22], miR-1 [33], and miR-133a [34]) and other non-cardiac miRNAs (miR-126, miR-146a, miR-30a, and miR-195) [20], [35]. However, the expressions of numerous circulating miRNAs in AMI are still unknown, and confirming the expression profile of circulating miRNAs in AMI is crucial for carrying out the functional researches of miRNAs in AMI.

MicroRNA-21-5p, located within the intronic region of the TMEM49 gene [36], has been reported to play a crucial role in cardiovascular system [37]. Recently, several studies confirmed that miR-21-5p was associated with ischemic heart disease. In an early study, van Rooij et al. found that the expression of miR-21-5p was increased in the border zone of infracted hearts in the late phase of AMI [38]. In other study, investigators found that the expression of miR-21-5p was significantly decreased in infracted areas and increased in border areas in rat hearts during the early phase of AMI. Furthermore, the protective effect of miR-21-5p on cell apoptosis induced by ischemia was confirmed both in vivo and in vitro [39]. These observations indicated that miR-21-5p may play critical roles in the early phase of AMI. However, the dynamic expression of circulating miR-21-5p in early phase of AMI is still unknown. MiR-361-5p is a cancer-related miRNA [40], [41], and miR-519e-5p is a placental-specific miRNA [42]. There are no previous reports evaluating the role of miR-361-5p and miR-519e in cardiovascular diseases. In our previous study, the aberrant expressions of circulating miRNA-21-5p, miRNA-361-5p and miRNA-519e-5p were observed in patients with coronary atherosclerosis by miRNA microarrays (manuscript under revision). In the present study, we aimed to confirm the expression of these three miRNAs in AMI, evaluate the ability of circulating miRNAs for diagnosing AMI, and investigate the cellular origin of these miRNAs. Two different cohorts were enrolled in this study. In the first cohort, we found that the plasma levels of miR-21-5p and miR-361-5p were markedly increased in patients with AMI compared to healthy control, whereas the expression of miR-519e-5p was reduced. These data are consistent with our previous results of miRNAs microarray. Furthermore, circulating miR-21-5p and miR-361-5p exhibited similar dynamic trends to plasma cTnI in the early phase of AMI. Both of them rapidly increased at first, achieved their peak at 16.4±1.5 hours (at 4 h after T0) after the onset of AMI symptoms, and then gradually declined in the following days. However, plasma miR-519e-5p maintained low level in the first 3 days of AMI. Next, the correlations between circulating miRNAs and cTnI were confirmed. The results indicated that two elevated miRNAs showed positive correlations with cTnI, whereas plasma miR-519e-5p exhibited a negative relationship with cTnI.

Furthermore, blood samples from 5 AMI patients who received emergency PCI were collected 1 week after they were admitted to the hospital for investigating the expressions of these miRNAs in plasma. The results showed that the expressions of three miRNAs approached their normal levels after 1 week.

In vitro, hypoxia experiment was performed to investigate the source of circulating miRNAs. Interestingly, following treatment with cobalt chloride for 24 hours, the expressions of miR-21-5p and miR-361-5p were significantly increased in both cardiomyocytes and their medium, whereas the level of miR-519e-3p was significantly reduced. The expressions of these three miRNAs in cardiomyocytes and culture medium after hypoxia were consistent with their expressions in plasma from AMI patients, which suggested that these circulating miRNAs may originate from injured cardiomyocytes in AMI under hypoxic conditions.

In addition, the expression of miR-519e-5p was significantly increased in peripheral blood neutrophils of AMI patients compared to control. Considering the high level of neutrophils in AMI, we suspect that the reduction of circulating miR-519e-5p in AMI may not only result from decreased expression in cardiomyocytes, but also from enhanced uptake by neutrophils in peripheral circulation.

To evaluate whether the three circulating miRNAs are specific to myocardial infarction, 9 IS patients and 8 patients with PE were recruited in the second cohort. The results showed that the expression of circulating miR-519e-5p in AMI was opposite to the other ischemic diseases. This result indicated that plasma miR-519e-5p is specific to AMI, and may be used as an effective biomarker to discriminate AMI from pulmonary embolism and ischemic stroke.

Heparin has been reported to selectively affect the quantification of circulating miRNAs [43]. Two blood samples were collected before and after the administration of heparin during PCI to detect the effects of heparin on these three miRNAs. The results showed that heparin did not have statistically significant effects on these miRNAs.

Receiver operating characteristic analysis was performed to investigate the abilities of the three miRNAs for distinguishing AMI patients from healthy volunteers. As expected, all of them showed strong diagnostic value for discriminating AMI patients from healthy controls in the early phase of AMI. Interestingly, the higher AUC values of miR-21-5p, miR-361-5p and miR-519e-5p appeared at T0, 4 h and 24 h. These results suggested that elevated plasma miR-21-5p and miR-361-5p were suitable as biomarkers for AMI in the early phases, whereas lowered circulating m-519e-5p was also appropriate. Furthermore, when combining these miRNAs together, the diagnostic accuracy became significantly raised with the AUC of 0.989 (95% confidence interval 0.000–1.000), 1.000 (95% confidence interval 0.000–1.000) and 0.995 (95% confidence interval 0.000–1.000) at T0, 4 h and 24 h, respectively. All these results suggested that circulating miR-21-5p, miR-361-5p and miR-519e-5p were strong and independent predictors for AMI.

Although elevated plasma miR-21-5p and miR-361-5p were not specific for cardiac ischemia, reduced circulating miR-519e-5p was sufficient to serve as a powerful predictor for differentiating AMI from other ischemic diseases.

Although our results are promising, there are several limitations in this study. Firstly, the numbers of participants are not enough for the in-depth analysis; secondly, only 5 blood samples were obtained in the follow-up investigation, and the follow-up period was also not long enough. Thirdly, the correlation between circulating miRNAs and troponin is not very positive, there are still lots of values which are either miRNA -high/cTnI-low or miRNA-low/cTnI-high. So a larger population is needed in future studies.

In summary, the present study provided first insights in measuring the dynamic changes of circulating miR-21-5p, miR-361-5p and miR-519e-5p levels in the early phase of AMI, and identified the cellular origin of these three miRNAs. Furthermore, we confirmed the levels of the three circulating miRNAs in patients with ischemic stroke and pulmonary embolism, and circulating miR-519e-5p was identified as a specific biomarker for cardiac ischemia. Finally, ROC analysis suggested that circulating miR-21-5p, miR-361-5p and miR-519e-5p can be novel and sensitive biomarkers for diagnosing AMI.

## Methods

### Ethics Statement

Experiments were conducted according to the Declaration of Helsinki. This study was supported and approved by the Ethics Committee of Tongji Hospital.

### Characteristics of participants and blood sample collection

In this study, a total of 83 participants involved in two cohorts were enrolled after obtaining written informed consents.

The first cohort contained 45 subjects, 17 of them had been documented as AMI, and other 28 healthy volunteers without evidence for CAD were selected as the control. The inclusion criteria for AMI patients were based on the recent definition [44]. Briefly, AMI patients were clinically diagnosed by the combination of the following criteria: 1) acute ischemic-type chest pain in last 24 hours; 2) electrocardiogram change (pathological Q wave, ST-segment elevation or depression); 3) elevated plasma cTnI (>0.1 ng/ml). In order to detect the expression trends of circulating miRNAs in early phase of AMI, 6 blood samples were acquired from AMI patients, the first blood sample (denoted by T0) was collected immediately from AMI patients after admission in Tongji hospital, and other subsequent blood samples were obtained at 4, 12, 24, 48 and 72 hours after T0, denoted by 4 h, 12 h, 24 h, 48 h and 72 h, respectively. Participants in this cohort were inpatients or outpatients from Tongji Hospital between October 2009 and May 2010.

The second cohort, including 9 AMI patients, 8 patients with pulmonary embolism, 9 ischemic stroke patients, and 12 healthy subjects, were used for investigating the levels of circulating miRNAs in various obstructive diseases, and evaluating whether these circulating miRNAs have abilities to discriminate AMI from other ischemic diseases. Blood samples were obtained from each patient after admission in Tongji hospital from October 2013 to March 2014. In order to assess the impact of heparin on circulating miRNAs, two blood samples were collected before and after the procedure of PCI during which heparin would be administrated.

Five milliliter blood samples were collected to EDTA anti-coagulant tube via venous puncture. After isolation by centrifugation, the plasma were transferred to RNase-free tubes and stored at -80°C until further processing.

### Cell Culture and Treatment

H9c2, Raw 264.7, and HUVEC cells were obtained from the American Type Tissue Collection (ATCC), and were cultured in Dulbecco's modified Eagle's medium (DMEM, Gibco, Grand Island, NY) or Roswell Park Memorial Institute (RPMI) 1640 medium (RPMI 1640, Gibco, Grand Island, NY) supplemented with 10% fetal bovine serum (FBS, Gibco, Grand Island, NY), respectively. Cells were grown at 37°C with an atmosphere of 5% CO2. For hypoxia experiments, cells were treated with 200 uM CoCl_2_ in 6 well plates. After treated with CoCl_2_ for 24 hours, cells and medium were collected for extracting the RNAs, respectively.

### Isolation of peripheral blood neutrophils

Peripheral blood neutrophils were isolated from all the AMI patients and 5 healthy volunteers in the second cohort using peripheral blood neutrophils extraction kit (TBDsciences, Tianjin, China) according to the manufacturer's protocol.

### RNA preparation

Total RNAs from plasma and medium were isolated by TRIzol LS Reagent (Invitrogen) according to the manufacturer's protocol as described previously [34]. TRIzol Reagent (Invitrogen) was used to extract RNAs from cells in accordance with the manufacturer's protocol.

### Detection and Quantification of miRNAs by real-time PCR

Two microgram of total RNA was reverse-transcribed using Transcript First-strand cDNA synthesis SuperMix (TransGen Biotech, Beijing, China) according to the manufacturer's protocol. Briefly, 50 µL reagents were incubated for 60 min at 42°C, 10 min at 70°C, and then preserved at 4°C. The Bulge-Loop miRNA qRT-PCR Detection Kit (Ribobio Co., Guangzhou, China) and SYBR Green PCR SuperMix Kit (TransGen Biotech, Beijing, China) were used in real-time PCR for examining the relative quantification of circulating miRNAs according to the manufacturer's protocol with the Rotor-Gene 6000 system (Corbett Life Science, QIAGEN, Hilden, Germany). U6 was measured as endogenous control for normalizing the date of experimental qRT-PCR. Each specimen was measured in triplicate. The threshold cycle (Ct) value was defined as the cycle number at which the fluorescence signals passed the fixed threshold. In our experiment, the detection limit of Ct value was defined as 40 [32], [33], [45].

### Cardiac Troponin I determination

The concentrations of plasma cTnI were measured in plasma samples of all the participants by the Human Troponin I ELISA kit (Abnova, Taiwan, China) according to the protocol of manufacturer.

### Statistical Analysis

Relative miRNAs expression levels were calculated by 2^−ΔΔct^ method. Values are expressed as mean ± SEM unless otherwise indicated. Student's t test (2 groups), ANOVA (n groups), Mann-Whitney U test (2 groups), Kruskal-Wallis test (n groups) were used as appropriate. Categorical variables were compared by χ^2^ test. Correlation analysis for normally distributed variables was performed using the Pearson correlation coefficient. Non-normally distributed variables were assessed by Spearman correlation analysis. The ROC curve was used to assess miRNAs as predictors for distinguishing AMI. Multiple logistic regression analysis was carried out for evaluating the combined diagnostic accuracy of circulating miRNAs. All statistical calculations were performed by SPSS 17.0 software, and all p-value are two-sided and p<0.05 was considered a statistically significant difference.

## Supporting Information

File S1 **Supporting information.** Figure S1, Diagnostic value of circulating miRNAs in AMI patients. the ROC curves of miR-21-5p for distinguishing AMI from healthy volunteers at T0 (A), 4 h (B) and 24 h (C), respectively; the ROC curves of miR-361-5p for distinguishing AMI from healthy volunteers at T0 (D), 4 h (E) and 24 h (F), respectively; the ROC curves of miR-519e-5p for distinguishing AMI from healthy volunteers at T0 (G), 4 h (H) and 24 h (I), respectively. Table S1, The treatment of 17 AMI patients in the first cohort.
(DOC)
