# Supplementary material for: Atherosclerosis-Related Circulating miRNAs as Novel and Sensitive Predictors for Acute Myocardial Infarction
Source: PLoS One. 2014 Sep 3;9(9):e105734. doi: 10.1371/journal.pone.0105734 (PMC4153586; doi:10.1371/journal.pone.0105734)
Supplement: File S1 — Supporting information. Figure S1, Diagnostic value of circulating miRNAs in AMI patients. the ROC curves of miR-21-5p for distinguishing AMI from healthy volunteers at T0 (A), 4 h (B) and 24 h (C), respectively; the ROC curves of miR-361-5p for distinguishing AMI from healthy volunteers at T0 (D), 4 h (E) and 24 h (F), respectively; the ROC curves of miR-519e-5p for distinguishing AMI from healthy volunteers at T0 (G), 4 h (H) and 24 h (I), respectively. Table S1, The treatment of 17 AMI patients in the first cohort. (DOC) [file pone.0105734.s001.doc]

**Supplemental Figure 1**

**Supplemental Table 1. T**he treatment of 17 AMI patients in the first cohort.

| **AMI patients** | **The time of admission in hospital** | **The door to balloon time** | **The detail of treatment** |
| --- | --- | --- | --- |
| Patient 1 (male) | 6 hours after the onset of AMI symptoms | - | Lysis therapy |
| Patient 2 (female) | 14 hours after the onset of AMI symptoms | - | Delayed PCI |
| Patient 3 (male) | 6 hours after the onset of AMI symptoms | 75 min | Emergency PCI |
| Patient 4 (male) | 6 hours after the onset of AMI symptoms | 125 min | Emergency PCI |
| Patient 5 (male) | 20 hours after the onset of AMI symptoms | - | Delayed PC |
| Patient 6 (male) | 17 hours after the onset of AMI symptoms | - | Delayed PC |
| Patient 7 (male) | 4 hours after the onset of AMI symptoms | 50 min | Emergency PCI |
| Patient 8 (female) | 16 hours after the onset of AMI symptoms | - | Delayed PC |
| Patient 9 (male) | 18 hours after the onset of AMI symptoms | - | conservative treatments |
| Patient 10 (female) | 16 hours after the onset of AMI symptoms | - | conservative treatments |
| Patient 11 (male) | 20 hours after the onset of AMI symptoms | - | Delayed PC |
| Patient 12 (male) | 16 hours after the onset of AMI symptoms | - | conservative treatments |
| Patient 13 (male) | 20 hours after the onset of AMI symptoms | - | conservative treatments |
| Patient 14 (male) | 10 hours after the onset of AMI symptoms | 80 min | Emergency PCI |
| Patient 15 (male) | 3 hours after the onset of AMI symptoms | - | Lysis therapy |
| Patient 16 (female) | 12 hours after the onset of AMI symptoms | - | conservative treatments |
| Patient 17 (female) | 7 hours after the onset of AMI symptoms | 90 min | Emergency PCI |
